# Perioperative management in acute and chronic spinal cord injury, narrative review

**DOI:** 10.1186/s44158-025-00252-z

**Published:** 2025-06-23

**Authors:** Israel Valdez-Resendiz, Estefany Nohemí Salgado-Camarillo, Fernanda Hernández-Morales, César Alejandro Martínez-de los Santos, Chiara Robba

**Affiliations:** 1 Department of Anesthesiology Hospital Zambrano Hellion TecSalud, Multicenter Program of Medical Specialties of the School of Medicine and Health Sciences of the Tecnológico de Monterrey, Monterrey, Nuevo Leon Mexico; 2 Departments of Anesthesia and Intensive Care, Policlinico San Martino IRCCS, for Oncology and Neuroscience, Genoa, Italy

**Keywords:** Spinal cord injury, Shock, Autonomic dysreflexia, Complications, Anesthesia, Perioperative care

## Abstract

Spinal cord injury (SCI) causes temporary or permanent changes and alterations in patients’ motor, sensory, or autonomic function, significantly impacting their quality of life and requiring clear goals and optimization of anesthesia and perioperative care for acute and chronic spinal cord injuries. SCI results from various etiologies and involving two principal pathophysiological mechanisms: primary and secondary injury. The first is result of the traumatic event, with irreversible neuronal damage, the second is generated as a consequence and in the minutes after the first and can continue for weeks or months causing degenerative damage to the spinal cord. It is in the secondary lesion where the objectives of anesthetic and perioperative management should be focused, especially in acute lesion. A conscientious and detailed preoperative evaluation allows to identify, injury level, evolution time, airway evaluation, cervical stability, hemodynamic status, ventilatory function and associated injuries must be determined. It is important to differentiate potential hemodynamic alterations and types of shock to prevent, especially in injuries greater than T6 and if necessary, provide early management in order to maintain adequate spinal cord perfusion. The objective of this review is to identify the pathophysiological mechanisms of spinal cord injury and the secondary systemic alterations and complications, as well as to establish specific optimization objectives during anesthetic management and perioperative care, which could reduce injury progression, prevent and control potential complications, and improve the quality of life of patients with this condition.

## Introduction

Spinal cord injury (SCI) is one of leading causes of morbidity and mortality worldwide [1–3].

It is defined as any damage to the spinal cord that causes changes and alterations in motor, sensory, or autonomic function, either temporarily or permanently. Its etiology can be traumatic (primarily motor vehicle accidents), accounting for 80% of cases, or non-traumatic (tumors, infections, degenerative diseases) [4].

This condition can result in adverse impact on the quality of life of patients due to the severity of autonomic, motor, and/or sensory dysfunction, then leading to alterations and potential complications in cardiovascular, respiratory, intestinal, urinary, musculoskeletal functions, and others, such as sexual dysfunction, adding to the emotional impact on patients [5].

The most frequently affected neurological level in traumatic spinal cord injuries is cervical region (16%–75%), followed by thoracic (16%–36%) and lumbar (9%–17%) regions, according to various studies [6].

There are two main pathophysiological mechanisms responsible for neuronal deterioration: the primary injury, which occurs at the time of trauma and is typically irreversible; and the secondary injury, which originates from various cellular and systemic mechanisms that follow the primary injury. This is the phase where prevention and agressive management can be implemented through specific goals and objectives in the acute SCI management approach [6].

Despite the current literature on spinal cord injury, which typically focuses more on surgical approach and management, there is currently little or no literature on clear objectives, guidelines, and specific and comprehensive recommendations for the perioperative management of this condition based on the type and purpose of the procedure, the timing of the intervention, and the level of the injury that guides the anesthesiologist in perioperative management and care [7].

The relatively low incidence makes it difficult to accumulate clinical experience or evidence from cohort studies, which complicates the establishment of robust recommendations. There is a lack of consensus regarding management, treatments, and standardization of care across centers that would lead to improved neurological recovery [8].

Recently, the AO Spine/Praxis Clinical Practice Guidelines for the Management of Acute Spinal Cord Injury have been published, focusing on various topics, including the timing of surgical decompression, the hemodynamic management of acute spinal cord injury, and the prevention, diagnosis, and treatment of intraoperative spinal cord injury [9].

These and other guidelines have various definitions, scales, management recommendations, monitoring, areas for future research, and challenges in their implementation [10]; however, there are few recommendations or approaches regarding anesthetic management and perioperative care beyond blood pressure targets for spinal cord perfusion that minimize injury and improve neurological prognosis. Therefore, the objective of this review is to identify and establish specific goals for the management of patients with acute or chronic spinal cord injury.

## Materials and methods

Narrative review based on a comprehensive and structured bibliographic search of peer-reviewed publications in Spanish and English in the Medline, PubMed, Embase, and Web of Science databases using combinations of the keywords and MeSH terms: Spinal cord injury; spinal shock; neurogenic shock; autonomic dysreflexia; secondary injury in spinal cord injury anesthesia and perioperative care.

Points of consensus and controversy were identified and described based on the available literature related to the study’s primary objective. The points of consensus were summarized, and two figures and three tables were prepared on the topic.

### Pathophysiology of SCI

The pathophysiological mechanisms of SCI are classified into primary and secondary injury. The primary injury results from the traumatic event, in this fase, typically neuronal tissue damage is irreversible. The mechanical forces responsible for primary injury in spinal cord trauma include section, traction, hyperextension, rotation, compression, and laceration, but the most common is compression; complete sectioning is a rare event [11].

**Fig. 1:**
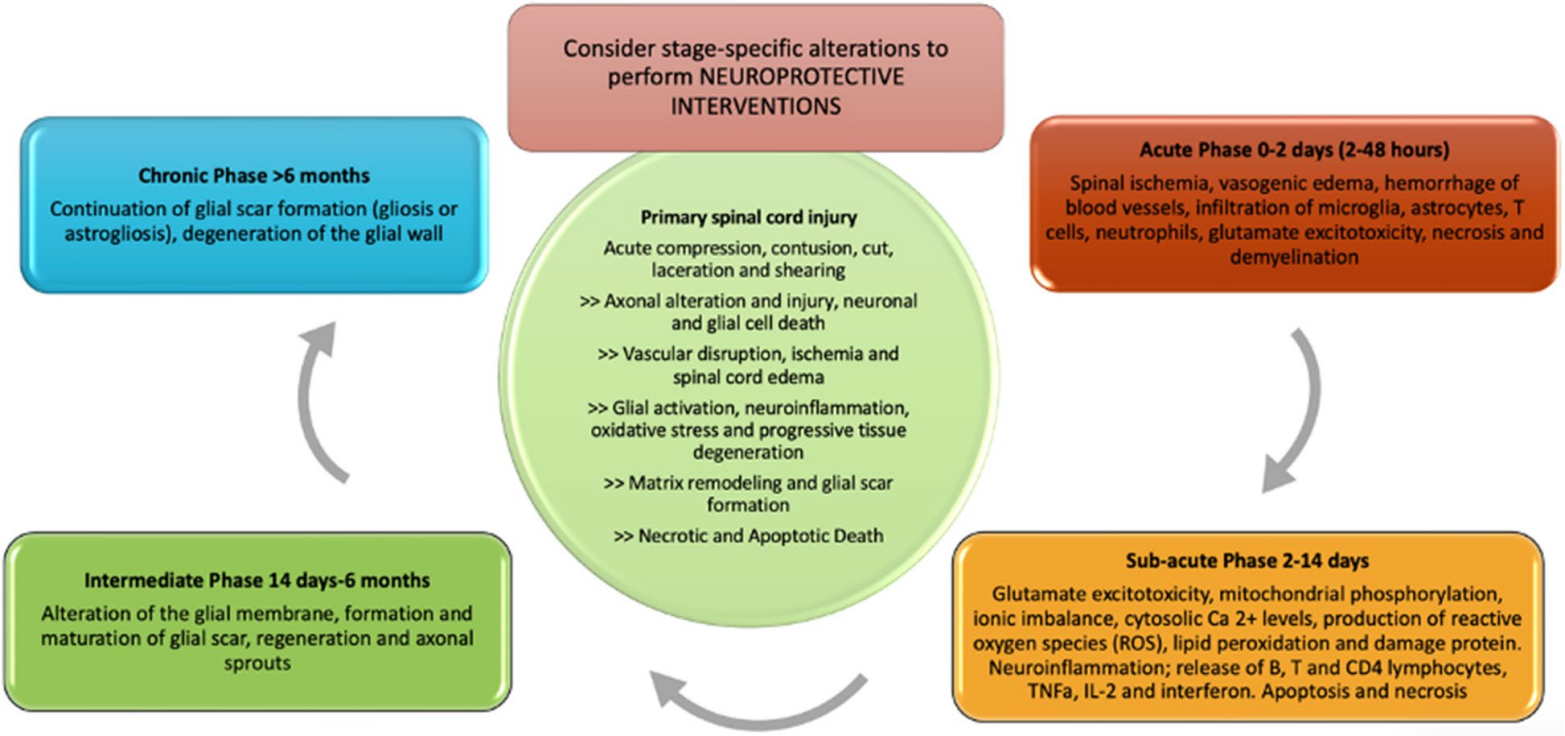
Pathophysiology of SCI and primary and secondary injury processes after traumatic spinal cord injury, classified according to its onset timing

The result of the primary injury mainly leads to the destruction of spinal cord tissue, vascular injury and disruption of cell membranes, ischemia, inflammation, release of free radicals and lipid peroxidation, excitotoxicity and necrotic and apoptotic death, further triggering a cascade of systemic and metabolic events that constitute the secondary injury. This secondary injury is a consequence of these events, beginning within minutes of the primary injury and potentially proceeding for weeks or months, causing degenerative damage to the spinal cord in the area of injury or surrounding tissue if not controlled [12]. It is classified according to its onset timing into acute (< 48 h), subacute (2–14 days), intermediate (14 days–6 months), and chronic (> 6 months) [13, 14] (Fig. 1).

The mechanisms of secondary injury include alterations in vascular flow, ionic imbalance, exocytosis, disturbances in calcium flow, edema, necrosis, lipid peroxidation, and free radical formation. If this continues into the subacute phase, neuronal apoptosis and axonal demyelination occur. The chronic phase of secondary injury is characterized by gliotic scar maturation [14, 15].

Systemically, cervical and thoracic spinal injuries can cause spinal shock or medullary shock due to the involvement of sympathetic roots, reducing vasomotor tone and causing bradycardia. This reduction in spinal cord perfusion exacerbates the existing ischemia or hypoperfusion. On the other hand, paralysis of the respiratory muscles causes ventilatory abnormalities, especially during the inspiratory phase, leading to hypoxia and hypercapnia [16].

### Classification

The most commonly used classification system of clinical evaluation and severity classification to determine the degree of neurological dysfunction during spinal cord injury is the International Standards for Neurological Classification of Spinal Cord Injury (ISNCSCI), better known as the ASIA Impairment Scale (AIS) [17], which is worth reviewing and keeping in mind at the time of evaluation. It accurately and specifically evaluates motor and sensory function. The motor examination evaluates dermatomes C5 to S1 and classifies each dermatome from grade 0 to 5, with a maximum score of 100. The sensory examination considers dermatomes C2 to S5, evaluating pain and light touch or fine tactile sensation [18]. In addition to classifying the level, SCI is defined as complete or incomplete depending on sacral preservation, which is considered when there is sensory or motor function in the most caudal sacral segments (S4-S5). The presence or absence of anal contraction and deep anal pressure should be evaluated, as this can indicate a complete spinal cord injury and be associated with a more unfavorable prognosis [15]. To stratify the degree of dysfunction, the AIS categorizes patients into groups from A with a complete injury, no motor or sensory function in the sacral segments to E with no motor or sensory deficits [17, 18], allowing for the identification of injury severity and level and predicting possible complications, especially dysautonomic alterations, enabling proper management preparation (Table 1).

**Table 1:** ASIA Impairment Scale Classification according to the International Standards for Neurological Classification of Spinal Cord Injury (ISNCSCI)

Grade	ASIA Impairment Scale Classification	Muscle motor function	Sensory function
A	Complete	None below the level of injury and sacral segments S4–5	None below the level of injury sacral segments S4–5
B	Sensory incomplete	None below the level of injury sacral segments S4–5	Preserved below the neurological level, with some sensitivity preserved in the sacral segments S4 and S5
C	Motor incomplete	Motor function is preserved at the most caudal sacral segments for voluntary anal contraction. Less than half of key muscle functions below the single NLI have a muscle grade ≥ 3	Sensory incomplete status (sensory function preserved at the most caudal sacral segments S4–S5)
D	Motor incomplete	Motor incomplete status as defined above, with at least half (half or more) of key muscle functions below the single NLI having a muscle grade ≥ 3	Preserved
E	Normal	The patient’s functions are normal. All motor functions are preserved	All sensory functions are preserved

### Physiological alterations after spinal cord injury

The main physiological alterations in patients with SCI are hemodynamic, ventilatory, and an increased risk of thromboembolism, among others, therefore, according to the temporality of the injury, clear management objectives must be established.

### Cardiorespiratory function

Depending on the level and type of injury, varying degrees of pulmonary and cardiovascular impairment may occur.

Lung impairments associated with the level of spinal cord injury, as well as varying degrees of pulmonary muscle impairment, will influence the need for different levels of ventilatory support (Table 2).

**Table 2:** Pulmonary alterations associated with the level of spinal cord injury

Injury level	Lung disorder	Bibliography
C1–C3 injury	Complete loss of inspiratory muscle function, mainly the diaphragm and external intercostals. These patients typically require continuous and permanent ventilatory support	Linn WS, Adkins RH, Gong H Jr, Waters RL. Pulmonary function in chronic spinal cord injury: a cross-sectional survey of 222 southern California adult outpatients. Arch Phys Med Rehabil. 2000 Jun;81(6):757–63. 10.1016/s0003-9993(00)90107-2. PMID: 10857520
C3–C5 injury:	Ventilation is required in the first days or weeks following the injury, with varying degrees of diaphragmatic function and inspiratory muscle activity, resulting in reduced lung volumes and limited pulmonary reserve. There are periods when the patient may not require ventilatory support or may only need nocturnal support	Schilero GJ, Bauman WA, Radulovic M. Traumatic Spinal Cord Injury: Pulmonary Physiologic Principles and Management. Clin Chest Med. 2018 Jun;39(2):411–425. 10.1016/j.ccm.2018.02.002. PMID: 29779599
C6–C8 injury	Diaphragmatic innervation is intact, compatible with spontaneous ventilation. However, abdominal muscles do not contribute to diaphragmatic function, impacting the inspiratory phase with reduced inspiratory volumes. These patients remain at risk for respiratory failure and pneumonia due to poor secretion control	Schilero GJ, Bauman WA, Radulovic M. Traumatic Spinal Cord Injury: Pulmonary Physiologic Principles and Management. Clin Chest Med. 2018 Jun;39(2):411–425. 10.1016/j.ccm.2018.02.002. PMID: 29779599
T1–T12 injury	Diaphragmatic function is intact with minimal loss of intercostal muscle strength, as well as abdominal muscle strength, allowing for independent spontaneous ventilation with minimal need for ventilatory support. Cough effectiveness depends on the thoracic level of the injury. Those with a lower-level SCI have improved secretion expulsion	Linn WS, Adkins RH, Gong H Jr, Waters RL. Pulmonary function in chronic spinal cord injury: a cross-sectional survey of 222 southern California adult outpatients. Arch Phys Med Rehabil. 2000 Jun;81(6):757–63. 10.1016/s0003-9993(00)90107-2. PMID: 10857520

In the acute phase, patients may present pulmonary edema that may be of cardiogenic origin (due to high levels of catecholamines and beta-endorphins) and/or non-cardiogenic (pulmonary hyperpermeability). Pulmonary thromboembolism and pneumonia may be complications that usually occur after the first 24 h. During the first year from injury, pulmonary parameters, including vital capacity, inspiratory capacity, total lung capacity, inspiratory and expiratory flows, and functional residual capacity, remain impaired [19, 20]. Furthermore, decreased chest compliance accompanies a reduction in pulmonary compliance, leading to microatelectasis formation, particularly during the first month post-injury. These pulmonary and systemic changes result in increased ventilatory work and muscle fatigue due to effort. An additional factor in this group of patients is the susceptibility to obstructive sleep apnea syndrome, with an incidence of up to 80% due to decreased airway muscle tone [19].

Cardiovascular mechanisms that occur after SCI with disruption of autonomic circuits include the following: disruption of the function of descending cardiovascular pathways responsible for sympathetic tone, leading to decreased vascular resistance and heart rate; decreased supraspinal sympathetic tone; and alterations in the peripheral neurovascular response that may cause vasodilation. Additionally, structural remodeling changes such as increased area and reduced wall thickness of the left ventricle occur, leading to altered cardiac contractility [21].

Spinal shock and neurogenic shock may occur together in the acute phase of SCI (24 to 72 h), presenting with bradycardia and hypotension, which typically require vasopressor support, along with loss of bladder control and sexual function [22]. The signs and symptoms of spinal and neurogenic shock appear simultaneously within the first 24 h; however, they represent two distinct entities (Table 3).

**Table 3:** Differences between spinal and neurogenic shock

	Spinal shock	Neurogenic shock	Bibliography
Definition	Flaccid paralysis with transient loss of reflexes and sensory-motor function below the level of injury	Loss of sympathetic tone with unopposed parasympathetic control, leading to vasodilation, decreased cardiac output, and cardiovascular instability	Biering-Sørensen F, Biering-Sørensen T, Liu N, Malmqvist L, Wecht JM, Krassioukov A. Alterations in cardiac autonomic control in spinal cord injury. Auton Neurosci. 2018 Jan;209:4–18. 10.1016/j.autneu.2017.02.004
Causes	Spinal cord injury mechanisms with flaccid paralysis from the level of injury (trauma, tumors, infections, autoimmune diseases)	Spinal cord injury (trauma, tumors, infections, autoimmune diseases) at or above T5. Spinal anesthesia, depression of the vasomotor center (e.g., intense pain, drugs, hypoglycemia)	Bustamante Bozzo R. Traumatismo raquimedular. Revista Chilena de Anestesia 2020: 50(1); 126–158. 10.25237/revchilanestv50n01-09 Biering-Sørensen F, Biering-Sørensen T, Liu N, Malmqvist L, Wecht JM, Krassioukov A. Alterations in cardiac autonomic control in spinal cord injury. Auton Neurosci. 2018 Jan;209:4–18. 10.1016/j.autneu.2017.02.004
Hemodinamic	No changes. Hypotension and bradycardia that may occur are secondary to neurogenic shock, which may occur simultaneously	Hypotension due to low systemic vascular resistance, excessive parasympathetic activity, and bradycardia with decreased venous return and cardiac output	Bustamante Bozzo R. Traumatismo raquimedular. Revista Chilena de Anestesia 2020: 50(1); 126–158. 10.25237/revchilanestv50n01-09 Alizadeh A, Dyck SM, Karimi-Abdolrezaee S. Traumatic Spinal Cord Injury: An Overview of Pathophysiology, Models and Acute Injury Mechanisms. Front Neurol. 2019 Mar 22;10:282. 10.3389/fneur.2019.00282. PMID: 30967837; PMCID: PMC6439316 Biering-Sørensen F, Biering-Sørensen T, Liu N, Malmqvist L, Wecht JM, Krassioukov A. Alterations in cardiac autonomic control in spinal cord injury. Auton Neurosci. 2018 Jan;209:4–18. 10.1016/j.autneu.2017.02.004
Associated autonomic symptoms	Difficulty breathing, intestinal and bladder dysfunction, priapism	Autonomic dysreflexia, orthostatic hypotension, temperature dysregulation. Dry and warm skin	Alizadeh A, Dyck SM, Karimi-Abdolrezaee S. Traumatic Spinal Cord Injury: An Overview of Pathophysiology, Models and Acute Injury Mechanisms. Front Neurol. 2019 Mar 22;10:282. 10.3389/fneur.2019.00282. PMID: 30967837; PMCID: PMC6439316 Biering-Sørensen F, Biering-Sørensen T, Liu N, Malmqvist L, Wecht JM, Krassioukov A. Alterations in cardiac autonomic control in spinal cord injury. Auton Neurosci. 2018 Jan;209:4–18. 10.1016/j.autneu.2017.02.004
Motor	Flaccid paralysis	Flaccid paralysis that may occur is secondary to spinal shock that occurs at the onset of primary injury	Yue JK, Winkler EA, Rick JW, Deng H, Partow CP, Upadhyayula PS, Birk HS, Chan AK, Dhall SS. Update on critical care for acute spinal cord injury in the setting of polytrauma. Neurosurg Focus. 2017 Nov;43(5):E19. 10.3171/2017.7.FOCUS17396
Reflexes	Areflexia or hyporeflexia in the early stage, hyperreflexia during the resolution stage	Areflexia or hyporeflexia that may occur is secondary to spinal shock at the onset of primary injury	Yue JK, Winkler EA, Rick JW, Deng H, Partow CP, Upadhyayula PS, Birk HS, Chan AK, Dhall SS. Update on critical care for acute spinal cord injury in the setting of polytrauma. Neurosurg Focus. 2017 Nov;43(5):E19. 10.3171/2017.7.FOCUS17396
Duration	From days to 4–6 weeks, up to a year	Generally resolves between the first and third week after the primary injury	Alizadeh A, Dyck SM, Karimi-Abdolrezaee S. Traumatic Spinal Cord Injury: An Overview of Pathophysiology, Models and Acute Injury Mechanisms. Front Neurol. 2019 Mar 22;10:282. 10.3389/fneur.2019.00282. PMID: 30967837; PMCID: PMC6439316 Yue JK, Winkler EA, Rick JW, Deng H, Partow CP, Upadhyayula PS, Birk HS, Chan AK, Dhall SS. Update on critical care for acute spinal cord injury in the setting of polytrauma. Neurosurg Focus. 2017 Nov;43(5):E19. 10.3171/2017.7.FOCUS17396 Biering-Sørensen F, Biering-Sørensen T, Liu N, Malmqvist L, Wecht JM, Krassioukov A. Alterations in cardiac autonomic control in spinal cord injury. Auton Neurosci. 2018 Jan;209:4–18. 10.1016/j.autneu.2017.02.004
Treatment	Stabilization and treatment of the underlying injury	Oxygen, fluids, and vasopressors with proper temperature control	Bustamante Bozzo R. Traumatismo raquimedular. Revista Chilena de Anestesia 2020: 50(1); 126–158. 10.25237/revchilanestv50n01-09 Alizadeh A, Dyck SM, Karimi-Abdolrezaee S. Traumatic Spinal Cord Injury: An Overview of Pathophysiology, Models and Acute Injury Mechanisms. Front Neurol. 2019 Mar 22;10:282. 10.3389/fneur.2019.00282. PMID: 30967837; PMCID: PMC6439316 Yue JK, Winkler EA, Rick JW, Deng H, Partow CP, Upadhyayula PS, Birk HS, Chan AK, Dhall SS. Update on critical care for acute spinal cord injury in the setting of polytrauma. Neurosurg Focus. 2017 Nov;43(5):E19. 10.3171/2017.7.FOCUS17396

Neurogenic shock should be suspected in SCI above T6 and is characterized by bradycardia and hypotension caused by parasympathetic effects with vagal activity without opposition, leading to bradycardia, reduced cardiac contractility, and hypotension, primarily due to damage to the sympathetic tracts at the cervical or upper thoracic level. It is secondary to sympathetic denervation of vascular smooth muscles, which leads to arterial vasodilation, blood pooling in the venous compartment, and interruption of sympathetic cardiac innervation (T1-T5), resulting in decreased vascular tone and resistance, reduced venous return, inadequate cardiac output, generalized and massive vasodilation, decreased tissue perfusion, and altered cellular metabolism [22, 23].

This phenomenon should not be confused with spinal shock, also known as medullary shock, which does not represent a shock state per se but rather a state of flaccid areflexia with complete loss of motor and sensory function below the level of the injury, accompanied by the loss of sphincter reflexes. It can occur after SCI between day 1 and day 4 in the acute and subacute period. Spinal shock typically lasts for days or up to 4 to 6 weeks, nevertheless this is not rule. After this period, the reflex activity of certain cutaneous and muscular groups progressively returns. The end of neurogenic shock is defined as the return of the bulbocavernosus reflexes that occurs in the first few days after the injury [23, 24].

The symptoms of spinal shock depend on the level. The most important ones are hemodynamic compromise and paralysis of the muscles that control ventilation.

Four phases of reflex recovery have been described, comprising the following progression [25]:

- Phase 1: Between 0 and 24 h, this phase is driven by motor neuron hyperpolarization and is characterized by areflexia or hyporeflexia. The first pathological reflex during this period is the delayed plantar reflex, followed by a bulbocavernosus, abdominal wall, and cremasteric reflexes. Sympathetic dysfunction can lead to bradyarrhythmias, atrioventricular conduction block, and hypotension.
- Phase 2: From day 1 to day 3, this phase is driven by denervation supersensitivity and receptor upregulation. Cutaneous reflexes are more prominent in this phase, while deep tendon reflexes remain absent.
- Phase 3: Between 4 days and 1 month, this phase is driven by synaptic and short axon growth. Deep tendon reflexes usually return in most patients, and the Babinski sign may appear.
- Phase 4: The duration of this phase ranges from 1 to 12 months and is driven by the growth of long axons and synapses. Cutaneous and deep tendon reflexes will be hyperactive even with minimal stimuli. Malignant hypertension and autonomic dysreflexia may also appear in this stage.

Spinal shock is a transient physiologic condition, typically improving within a few days to weeks. Therefore, management is not necessarily treating spinal shock but rather mitigating spinal cord damage and preempting secondary complications. This primarily consists of hemodynamic and respiratory stability to prevent further neurogenic injury and supportive therapy.

### Autonomic dysreflexia (AD)

Autonomic dysreflexia (AD) occurs in patients with SCI above T6 and is defined as episodes of severe hypertension resulting from a massive sympathetic discharge. To confirm AD, there must be an increase in systolic blood pressure of 20% above baseline, which may be accompanied by headache, diaphoresis, piloerection, and facial flushing [26]. It occurs due to supraspinal inhibition that restricts sympathetic outflow, in addition to destruction of the descending vasomotor pathways. Symptoms occurring above the level of injury are caused by vasodilation of the corresponding vessels in response to sympathetic inhibition, while symptoms occurring below the level of injury are caused by sympathetic stimulation and vasoconstriction [24–27].

AD may appear in the acute phase; however, it is much more common in patients with chronic SCI, particularly between the 3rd and 6th months, a time when patients are more prone to urological procedures.

AD episodes tend to decrease one year after spinal cord injury; however, they can occur if the patient is exposed to a trigger [28]. It is important to identify patients at higher risk of dysautonomia due to high-level spinal cord injuries and to remain vigilant for the appearance of signs and symptoms of excessive sympathetic activity, such as facial flushing, arrhythmias, sweating above the level of the injury in response to stimuli below the level of the injury, secondary to an increase in inhibitory impulses from the vasomotor centers.

This mechanism compensates to mitigate the vasoconstriction resulting from AD. AD can be triggered by any type of nociceptive stimulus such as bladder catheterization, abdominal distention, fecal impaction, compression, or surgical stimulation, so it is essential to identify and resolve the potential cause [28]. Once confirmed, it is important to elevate the patient's head to 30°, stop surgical or triggering stimuli, and in cases of significant hypertension, ensure adequate analgesia and anesthetic plane. If necessary, beta-blockers or short-acting vasodilators should be considered for infusion to avoid severe complications such as seizures, subarachnoid hemorrhage, myocardial infarction, or death [24].

### Temperature regulation

Temperature control is generally impaired, especially if the injury is at the cervical level. This occurs due to sensory dysregulation of thermoregulatory centers, reducing sympathetic vascular tone and sweating below the level of the injury [28, 29].

Positioning, as well as temperature control, plays a crucial role, as these patients are at high risk of developing pressure ulcers, and exposing these areas could trigger an episode of autonomic dysreflexia. Vasodilation below the level of the injury predisposes to hypothermia, which, coupled with impaired sweating, alters heat dissipation. Therefore, monitoring and maintaining temperature is essential [26].

### Thromboembolism

The risk of thromboembolism during the first 3 months after injury is approximately 85%. After this period, the risk decreases, and routine prophylaxis is no longer recommended. This is because, after this period, recovery of lower body muscle spasms occurs, along with the function of the muscle pump in muscles supplied by the femoral artery, and a reduction in venous distensibility, which reduces the generation of thrombi [30].

### Genitourinary alterations

Genitourinary alterations are the most common procedures requiring anesthesia in chronic SCI. They result from alterations in the motor and sensory innervation of the bladder, leading to neurogenic disease. There is a reduction in bladder volume as well as incomplete emptying. On the other hand, the main source of sepsis in SCI patients is the urinary tract [27–29].

### Gastrointestinal alterations

There is often a decrease and delay in gastric emptying, particularly in high-level injuries (> T6), caused by reflux, constipation, abdominal distention, and abdominal pain. Gallstone formation is more frequent than in the general population, which puts these patients at high risk for biliary complications [27–29].

### Spasticity and contractures

Spasticity occurs as a result of hyperexcitability of spinal reflexes. Contractures are also common and may complicate optimal positioning during surgery. Additionally, denervated muscles cause an increase in acetylcholine receptors, so when administering depolarizing neuromuscular blockers, this depolarization will occur throughout the muscle mass, raising serum potassium concentrations and increasing the risk to cardiac function [27, 28].

### Anesthetic management of patients with SCI acute or chronic

**Fig. 2:**
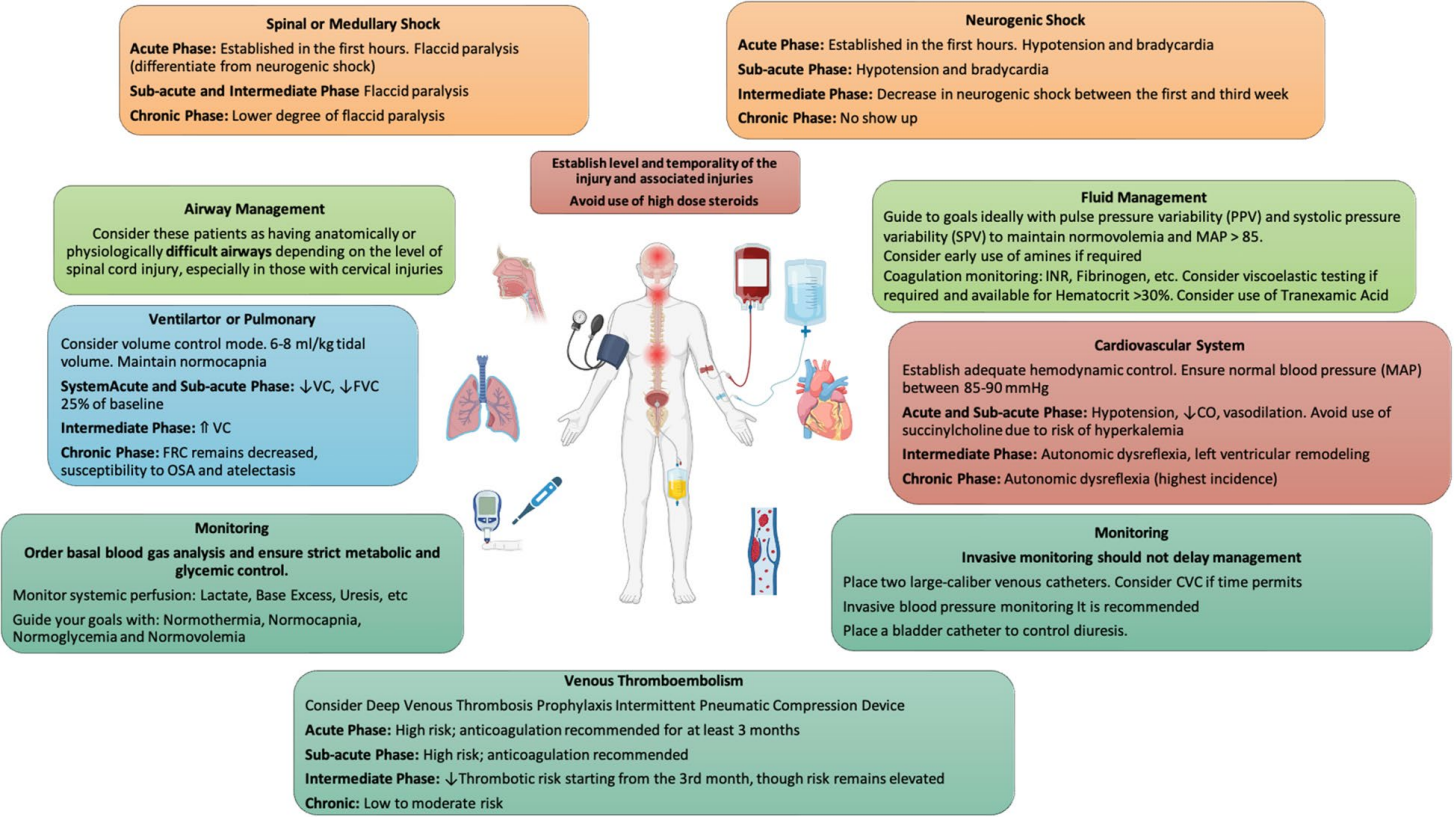
Systemic approach to acute and chronic spinal cord injury and main management objectives in the perioperative period. CO (cardiac output); VC (vital capacity); FVC (forced vital capacity); FRC (functional residual capacity); OSA (obstructive sleep apnea syndrome); CVC (central venous catheter)

The management of patients with acute or chronic spinal cord injury (SCI) presents a significant challenge for those unfamiliar with this pathology. The evaluation and approach to these patients require the cooperation and teamwork of a multidisciplinary team with specific goals to minimize the possibility of complications, improve their evolution, and their neurological and systemic prognosis [31, 32]. For optimal anesthetic and perioperative management, clear and well-defined goals must be established based on the type and objective of the procedure, the timing, and the level of the injury (Fig. 2, Table 4).

**Table 4:** Considerations according to the type of anesthesia

Type of anesthesia	Advantages	Disadventages	Bibliography
Spinal	Fewer episodes of dysautonomia in patients with chronic SCI. Most commonly used for urological procedures	Inability/difficulty in assessing the spread and level of the block	Zhang Y, Al Mamun A, Yuan Y, Lu Q, Xiong J, Yang S, Wu C, Wu Y, Wang J. Acute spinal cord injury: Pathophysiology and pharmacological intervention. Mol Med Rep. 2021 Jun;23(6):417. 10.3892/mmr.2021.12056. Epub 2021 Apr 13. PMID: 33846780; PMCID: PMC8025476 Yue JK, Winkler EA, Rick JW, Deng H, Partow CP, Upadhyayula PS, Birk HS, Chan AK, Dhall SS. Update on critical care for acute spinal cord injury in the setting of polytrauma. Neurosurg Focus. 2017 Nov;43(5):E19. 10.3171/2017.7.FOCUS17396
Epidural	Fewer episodes of dysautonomia; more useful in the perioperative period for pain control	Inability/difficulty in assessing the spread and level of the block	Yue JK, Winkler EA, Rick JW, Deng H, Partow CP, Upadhyayula PS, Birk HS, Chan AK, Dhall SS. Update on critical care for acute spinal cord injury in the setting of polytrauma. Neurosurg Focus. 2017 Nov;43(5):E19. 10.3171/2017.7.FOCUS17396
General anesthesia	Recommended for patients with acute SCI. Invasive monitoring recommended	Use of succinylcholine is contraindicated 72 h to 6 months post-acute event	Altaf F, Griesdale DE, Belanger L, Ritchie L, Markez J, Ailon T, Boyd MC, Paquette S, Fisher CG, Street J, Dvorak MF, Kwon BK. The differential effects of norepinephrine and dopamine on cerebrospinal fluid pressure and spinal cord perfusion pressure after acute human spinal cord injury. Spinal Cord. 2017 Jan;55(1):33–38. 10.1038/sc.2016.79

### Perioperative evaluation

The pre-anesthetic evaluation should be carried out immediately and as thoroughly as the urgency of the situation allows, including neurological assessment, level of injury, associated pathologies, duration of injury, medication use, anesthetic history, allergies, and airway evaluation. In acute SCI, associated injuries such as traumatic brain injury (TBI), fractures, or abdominal trauma should also be assessed, along with routine baseline laboratory tests, electrocardiogram, and ideally, a chest X-ray. It is recommended to obtain a baseline arterial blood gas upon entering the operating room to optimize the ventilatory and/or metabolic conditions required, including hematocrit monitoring and, if necessary, the need for blood transfusions. For patients with chronic SCI (> 6 months), the scheduled procedure, injury level, ventilatory and circulatory compromise, and history of dysautonomia should be considered [31].

The choice of anesthetic technique largely depends on the patient’s hemodynamic and ventilatory conditions, the scheduled procedure, and the patient’s ability to cooperate. In both acute and chronic SCI patients, general anesthesia is commonly used. However, for procedures on peripheral sites or the lower trunk, neuraxial and/or regional anesthesia is recommended, as it is well-tolerated and offers advantages such as greater hemodynamic stability, less manipulation of the airway, and a reduced need for mechanical ventilation. Currently, there are no studies comparing inhaled agents versus intravenous agents, and insufficient data exists to claim that one technique is superior to the other [33].

### Airway management

Any patient with SCI should be considered to have a cervical injury until proven otherwise, and difficult airway (DA) should be anticipated, especially during an acute event, as it may cause a new spinal cord injury or exacerbate an existing neurological deficit. It is advisable to have trained operators and available airway management equipment, including airway guides, supraglottic rescue devices, video laryngoscopes, flexible fiberoptic scopes, and even be prepared for emergency front-of-neck-access [33, 34]. Airway management in the presence of acute cervical spine injury remains a challenge, and the method of intubation in the traumatic context remains debated [34, 35].

Traditionally, awake fiberoptic bronchoscopy (FOB) has been recommended as it limits cervical spine movement during intubation and allows neurological examination after the procedure. However, this technique requires significant learning and skill, so it is advisable to have an expert in airway management and the specific device. Due to the widespread availability of video laryngoscopy (VL) with good results in these patients, the use of FOB has drastically decreased [34, 35]. In certain centers and reviews, VL is the most commonly used initial airway management technique, followed by FOB alone or combined with VL, and less frequently awake FOB or direct laryngoscopy. All techniques are associated with high success rates on the first attempt and no subsequent neurological injury [35].

It is important to avoid the use of succinylcholine after 48 h of spinal cord injury due to the risk of hyperkalemia and arrhythmias [33]. Routine cricoid pressure is not recommended, as it does not improve visualization of the vocal cords or increase intubation success.

Since all airway interventions involve some degree of cervical spine movement, precautions should be taken to limit neck movement using manual inline stabilization and a rigid cervical collar (which may increase intubation difficulty), as well as using the intubation technique in which the operator feels most comfortable and skilled [33–36]. Until recently, there were few evidence-based guidelines for physicians to support the safe and effective management of airways in this setting. Recently, multidisciplinary working groups of experts from various societies have published guidelines with recommendations to improve airway management in patients with suspected or confirmed cervical spine injury [36].

### Hemodynamic monitoring/management

Hemodynamic management is known to be one of the few available therapeutic options likely to improve neurological outcomes in patients with acute traumatic spinal cord injury. Increasing mean arterial pressure (MAP) aims to improve blood perfusion and oxygen delivery to the injured spinal cord to minimize secondary ischemic damage to neural tissue. Therefore, hemodynamic management may be important in the early stages of SCI to optimize blood flow to the tissue surrounding the primary lesion at risk of infarction [37].

In the spinal cord, autoregulation mechanisms function similarly to those of the brain, meaning that blood flow autoregulation is maintained at a mean arterial pressure (MAP) of 60 to 150 mmHg. However, these values can have significant interindividual variability, especially in hypertensive patients, where the autoregulation curve is shifted to the right and blood pressure target requirements are higher. Furthermore, this autoregulation is lost in the first 30 min after SCI, where blood flow remains constant and is compromised at MAP values below 60 mmHg or above 150 mmHg, putting spinal cord perfusion at risk during hypotension or spinal shock [12]. Therefore, there is controversy regarding interindividual variability, the benefits of blood pressure management, its actual impact on spinal cord perfusion, and its benefits and impact on neurological outcome.

The literature does not provide high-quality evidence to support a particular protocol, so currently, there is a growing interest in monitoring spinal cord perfusion pressure (SCPP), (difference between MAP and intrathecal pressure or intraspinal pressure) by invasive methods [38]. The 2013 American Association of Neurological Surgeons/Congress of Neurological Surgeons guidelines recommend maintaining MAP at 85–90 mmHg for 7 days after SCI to potentially improve outcomes [39]. However, ideally, SCPP should be considered in addition to MAP, including measurement of intrathecal pressure using a lumbar intrathecal catheter.

It is advisable to avoid hypotension and excessive hypertension, as well as proactively monitoring the timing and level of hypotension because hypoperfusion and hypoxemia worsen secondary injury mechanisms. Currently, it is suggested to increase mean arterial pressure to 75–80 mmHg as a lower limit, but not to actively increase it beyond an upper limit of 90–95 mmHg, to optimize spinal cord perfusion in acute traumatic SCI (Quality of evidence: very low; strength of recommendation: weak). This increase in MAP is suggested for 3 to 7 days to optimize spinal cord perfusion in acute SCI (quality of evidence: very low; strength of recommendation: weak) [37].

It is advisable to maintain normovolemia and use goal-guided fluid therapy, as well as the early use of vasoactive medications to avoid excessive fluid administration in order to prevent hypotension (systolic blood pressure < 90 mmHg), hypoperfusion and ischemia of the injured spinal cord, as well as secondary damage after SCI [40]. Several studies suggest that norepinephrine is better for maintaining MAP targets in these patients, with fewer arrhythmic events compared to dobutamine, and better spinal cord perfusion pressure [41].

Due to a lack of physical activity, coronary artery disease, atherosclerosis, hyperlipidemia, and insulin resistance are common in patients with chronic SCI. Furthermore, mortality is higher in patients over 60 years of age due to impaired cardiac and respiratory function and a higher incidence of cervical injury. Life expectancy depends on the severity, level of injury, and age, ranging from 1.5 years for mechanically ventilated patients over 60 years old with any level of injury to 50 years for 20-year-olds with preserved motor function. Fatal complications include thromboembolic disease and sepsis due to pneumonia, urinary tract infections, or pressure ulcers [42, 43], therefore, depending on the patient’s age and duration of the injury, we must take into account the corresponding considerations for this type of patient.

### Steroids in acute spinal cord injury

The use of corticosteroids following acute spinal cord injury is a controversial topic [44] and remains a common practice worldwide across different specialties in these types of injuries [45].

The NASCIS trials recommended high-dose methylprednisolone (MPS) administration for 48 h; however, the methodological quality of NASCIS studies has been widely questioned, with recommendations for categorizing these studies at evidence level III. Consequently, clinical recommendations and selectivity for their use have changed over the years [46, 47].

A Cochrane meta-analysis in 2016 demonstrated that high doses of MPS produced significant motor function recovery without increasing mortality if administered within 8 h of injury [48]. However, a new meta-analysis with new studies published showed that MPS was not associated with long-term neurological recovery [49].

The GRADE evidence-based clinical guidelines showed that no differences in motor recovery were found between patients treated with MPS and those who did not receive treatment. When MPS was administered within 8 h post-injury, combined results at 6 and 12 months indicated modest improvements in motor recovery in the treated group compared to the control group. There were no statistical differences in complications between the treated and untreated group [50].

Since 2013, various guidelines and societies [39, 50–53] do not recommend the use of high-dose intravenous methylprednisolone, due to the lack of level I or II evidence regarding its clinical benefit, while level I, II and III evidence indicates a possible association with harmful side effects and possible complications [3, 54]. Currently, high-dose MPS infusions are not recommended in patients presenting with an acute spinal cord injury after 8 h (moderate quality of evidence; weak strength of recommendation) [50]. Based on current evidence, widespread use of MPS is not recommended; however, it may still be used in some centers in specific cases, with careful consideration of the individual risk–benefit ratio and timing of the injury. Future, robust, randomized clinical studies are needed to clarify whether corticosteroids should be recommended for acute SCI.

## Conclusions

Rather than defining a particular type of anesthesia, the anesthesiologist should identify the level of injury, time of progression, airway status, cervical stability, hemodynamic status, ventilatory function, and associated injuries, to establish specific and well-defined systemic monitoring and management objectives in the perioperative period, as well as to prevent and control possible complications.

Potential hemodynamic alterations and types of shock must be differentiated in order to prevent and, if necessary, provide early treatment with the aim of maintaining adequate spinal cord perfusion. In acute spinal cord injury, potential complications in airway management must be prevented and the progression of secondary injury limited with specific and well-defined management objectives. In chronic spinal cord injury, the level and timing of the injury must be established to minimize the possibility of hemodynamic variations and early management if it occurs.

A systemic approach to acute and chronic spinal cord injury is recommended, with clear goals for anesthetic management and perioperative care, including normothermia, normoxia, normocapnia, normoglycemia, and normovolemia through fluid management, airway management, ventilation strategies, monitoring and hemodynamic support that allows for a target mean arterial pressure to maintain adequate spinal cord perfusion. It is advisable to establish institutional guidelines and protocols through a multidisciplinary and collaborative approach and management, including surgical staff, anesthesiologists, critical and neurocritical care physicians, neurophysiologists, physiatrists, physical therapists, and rehabilitation specialists.

The establishment of clinical trials, systematic reviews, multicenter studies, and consensus on the goals of perioperative systemic management protocols, as well as adequate follow-up, rehabilitation, and the study of pharmacological agents and therapeutic approaches with neuroprotective properties, could improve the outcomes of patients with spinal cord injuries. Each step in our management can have long-term implications and impact the quality of life of patients with this type of pathology.

### Study limitations

As a narrative review, the methodological nature of this study may present several limitations in the literature search, excluding several relevant articles on the topic with a more in-depth discussion and review focus.

## Data Availability

No datasets were generated or analysed during the current study.
